# The efficacy and safety of lenalidomide plus dexamethasone in relapsed and/or refractory multiple myeloma patients with impaired renal function

**DOI:** 10.1002/cncr.25139

**Published:** 2010-05-24

**Authors:** Meletios Dimopoulos, Adrian Alegre, Edward A Stadtmauer, Hartmut Goldschmidt, Jeffrey A Zonder, Carlos M de Castro, Zvenyslava Masliak, Donna Reece, Marta Olesnyckyj, Zhinuan Yu, Donna M Weber

**Affiliations:** 1 Department of Clinical Therapeutics, University of Athens School of MedicineAthens, Greece; 2 Hematology Department, Princess University HospitalMadrid, Spain; 3 Abramson Cancer Center, University of PennsylvaniaPhiladelphia, Pennsylvania; 4 Department of Internal Medicine V, University of HeidelbergHeidelberg, Germany; 5 Karmanos Cancer Institute, Wayne State UniversityDetroit, Michigan; 6 Department of Medical Oncology, Duke University Medical CenterDurham, North Carolina; 7 Institute of Blood Pathology and Transfusion Medicine, Hematology Department of the Academy of Medical Sciences of UkraineLviv, Ukraine; 8 Division of Hematology-Oncology, Princess Margaret HospitalToronto, Ontario, Canada; 9 Clinical Research and Development, Oncology, Celgene CorporationSummit, New Jersey; 10 Department of Lymphoma and Myeloma, The University of Texas M. D. Anderson Cancer CenterHouston, Texas

**Keywords:** renal impairment, multiple myeloma, lenalidomide, creatinine clearance, dexamethasone

## Abstract

**BACKGROUND:**

In patients with multiple myeloma, renal impairment (RI) at the time of diagnosis is associated with poor survival. To the authors' knowledge, the current retrospective analysis presented is the first to assess the impact of various degrees of renal dysfunction on safety and efficacy outcomes in a large cohort of patients with relapsed and/or refractory multiple myeloma who received treatment with lenalidomide plus dexamethasone.

**METHODS:**

Three hundred fifty-three patients from 2 large phase 3 trials were randomized to receive lenalidomide (25 mg) plus dexamethasone (40 mg). For the purpose of this analysis, RI was defined according to the calculated creatinine clearance (CL_Cr_) level as follows: mild or no RI (CL_Cr_ ≥ 60 mL/minute), moderate RI (CL_Cr_ from ≥ 30 mL/minute to <60 mL/minute), and severe RI (CL_Cr_ <30 mL/minute).

**RESULTS:**

The RI subgroups did not differ significantly in terms of the overall response rate (range, 50%-64%) or response quality (very good partial response or better, 27%-37%). In all RI subgroups, the time to progression and progression-free survival did not differ significantly compared with the mild or no RI group. Patients with RI experienced an increased incidence of thrombocytopenia, required more frequent lenalidomide dose reduction or interruption, and had shorter overall survival than patients with mild or no RI (*P* = .006). Lenalidomide plus dexamethasone led to improvement in renal function in the majority of patients.

**CONCLUSIONS:**

The results from this study indicated that, with careful monitoring of the CL_Cr_ level and adverse events as well as appropriate dose adjustments, lenalidomide plus dexamethasone is an effective and well tolerated treatment option for patients with multiple myeloma who have RI. Cancer 2010. © 2010 American Cancer Society.

Approximately 20% of patients with newly diagnosed multiple myeloma (MM) present with renal failure.^1^ Moreover, this end-organ damage is the second most common cause of death in patients with MM.^2^ Cast nephropathy, the most common cause of myeloma-associated renal injury, occurs in at least 30% of patients.^2^,^3^ Other causes of renal impairment (RI) in patients with MM include acute tubulopathy, amyloid light-chain amyloidosis, light-chain deposition disease, tubulointerstitial nephritis associated with monotypic light-chain deposits, and plasma cell tumor nodules.^2^ In addition, hypercalcemia, which may impact renal function, occurs in 33% of newly diagnosed patients.^4^-^6^ The degree of RI is usually mild to moderate (ie, plasma creatinine >1.3 mg/dL and <2 mg/dL) in approximately 50% of patients; however, severe RI (plasma creatinine ≥2 mg/dL) may be observed in 15% to 20% of patients at diagnosis.^3^,^5^-^7^

Effective myeloma control and resultant reduction in urinary light-chain excretion can lead to improvement in RI in 50% to 60% of patients.^3^,^6^,^8^,^9^ Although patients with MM who have persistent RI often have inferior survival, recovery of renal function, often made possible through novel drugs that better control the disease, is associated with an improvement in outcome.^4^ In patients with RI, drugs known as nephrotoxic should be avoided whenever possible. Medications that are excreted renally typically need dose and/or schedule adjustment to reduce the risk of side effects.^10^,^11^

Lenalidomide combined with dexamethasone is an effective treatment for MM that provides an overall response (OR) rate of 60% with a complete response (CR) rate of 15%, in patients with relapsed and/or refractory MM.^12^,^13^ Two-thirds of lenalidomide is eliminated unchanged through urinary excretion.^14^ To our knowledge, the retrospective analysis presented here is the first to assess the impact of various degrees of renal dysfunction on safety and efficacy outcomes with lenalidomide, such as OR, time to progression (TTP), progression-free survival (PFS), overall survival (OS), and improvements in renal function, in a large cohort of patients with relapsed and/or refractory MM who received lenalidomide.

## MATERIALS AND METHODS

In total, 704 patients who met the entry criteria were enrolled in MM-009 and MM-010, 2 large, phase 3, randomized, multicenter clinical trials that compared treatment with lenalidomide (25 mg daily on Days 1-21 of each 28-day cycle) plus dexamethasone (40 mg on Days 1-4, 9-12, and 17-20 every 28 days for 4 cycles and 40 mg on Days 1-4 every cycle thereafter) versus dexamethasone alone (identical schedule).^12^,^13^ Treatment was continued until patients developed either unacceptable toxicity or disease progression. Toxicity was graded according to the National Cancer Institute Common Toxicity Criteria version 2.0, as reported previously.^12^,^13^

Three hundred fifty-three patients who were randomized to receive lenalidomide plus dexamethasone in these 2 trials^12^,^13^ were included in the current subgroup analysis. Inclusion and exclusion criteria were published previously.^12^,^13^ Entry criteria for renal function were based on serum creatinine levels. All patients were required to have a baseline serum creatinine level <2.5 mg/dL. Renal function was assessed throughout the course of the study by measuring serum creatinine levels. For the purpose of the present RI analysis, renal function was assessed by using the serum creatinine level to calculate creatinine clearance (CL_Cr_) with the Cockroft-Gault equation. Then, patients were subdivided into RI subgroups based on their CL_Cr_ values, defined as follows: mild or no RI, CL_Cr_ ≥60 mL/minute; moderate RI, CL_Cr_ ≥30 mL/minute and <60 mL/minute; or severe RI, CL_Cr_ <30 mL/minute.^15^

The objective of the current analysis was to evaluate the efficacy of therapy, safety, and tolerability with lenalidomide plus dexamethasone in patients who had mild or no RI compared with patients who had moderate and severe RI. Response to treatment was assessed according to the modified European Group for Blood and Marrow Transplantation criteria for CR and partial response (PR), according to criteria described by the International Myeloma Working Group for a very good PR (VGPR).^16^,^17^ TTP was measured from the date of randomization to the date of the first assessment that identified disease progression. Patients who died or discontinued the study without evidence of disease progression were censored at the last evaluation for assessment of TTP. PFS was measured from the date of randomization to the date of the first assessment that identified disease progression or the date of death during treatment, whichever occurred first. Patients who were alive and discontinued study therapy without evidence of disease progression were censored at the last evaluation for assessment of PFS. OS was measured from the date of randomization to the date of death from any cause or was censored at the last follow-up.

Data on OR (CR + VGPR + PR), TTP, and PFS were assessed up to unblinding, which occurred in June 2005 for MM-009 and August 2005 for MM-010, for a median follow-up duration of 17.5 months. The cutoff for follow-up data on OS was January 2007, with a median follow-up duration of 31.3 months.

Differences in OR rates were analyzed using continuity-corrected Pearson chi-square tests. Time-to-event variables with censoring, including TTP, PFS, and OS, were estimated by using the Kaplan-Meier method. Two-sided log-rank tests were used for comparisons of TTP, PFS, and OS.

## RESULTS

### Baseline Characteristics

Of the 353 patients who were randomized to receive lenalidomide plus dexamethasone, baseline CL_Cr_ data were available for 341 patients. Of these patients, 243 (71%) had mild or no RI (CL_Cr_ ≥60 mL/minute), 82 (24%) had moderate RI (CL_Cr_ ≥30 mL/minute and <60 mL/minute), and 16 (5%) had severe RI (CL_Cr_ <30 mL/minute). According to protocol exclusion criteria, all patients who were included in this analysis had a relatively low serum creatinine level (<2.5 mg/dL), although 16 patients had severe RI. Patients who had RI were older, more likely to be women, and had higher serum β_2_-microglobulin levels compared with patients who did not have RI (all *P* < .05) (Table 1). Besides this, renal function subgroups were balanced (Table 1).

**Table 1:** Baseline Characteristics of Patients

Characteristic	Mild or No RI: CL_Cr_ ≥60 mL/min	Moderate RI: CL_Cr_ ≥30 mL/min to <60 mL/min	Severe RI: CL_Cr_ <30 mL/min
No. of patients	243	82	16
Median age (range), y	61 (33-82)	69 (38-84)^a^	73 (56-86)^a^
Men, %	67	43^a^	25^a^
ECOG score 0 or 1, %	89	87	69
Median time since diagnosis (range), y	3.1 (0.4-15.7)	3.4 (0.5-14.6)	3.4 (1.0-8.7)
Bone lesions, %	75	72	69
Serum β_2_-microglubulin >2.5 mg/L, %	62	89^a^	100^a^
Immunoglobulin G, %	66	63	56
Immunoglobulin A, %	21	20	19
Durie-Salmon stage III, %	62	72	75
No. of prior lines of therapy, median (range)	2 (0-8)	2 (0-5)	2 (0-3)
Prior therapies, %			
Thalidomide	35	33	56
Bortezomib	9	6	0

RI indicates renal impairment; CL_Cr_, creatinine clearance; ECOG, Eastern Cooperative Oncology Group.

a *P* < .05 versus patients with mild or no RI.

### Efficacy

**Figure 1:**
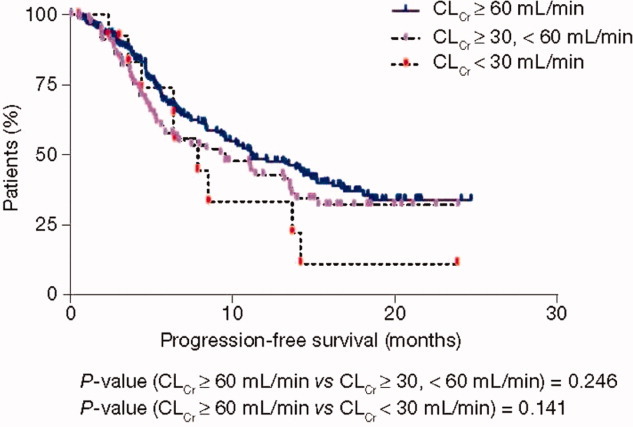
This is a Kaplan-Meier plot of progression-free survival according to renal impairment (RI). RI was defined by creatinine clearance (CL_Cr_) level as mild or no RI (CL_Cr_ ≥60 mL/minute), moderate RI (CL_Cr_ from ≥30 mL/minute to <60 mL/minute), or severe RI (CL_Cr_ <30 mL/minute).

There were no statistical differences in OR rates between the 3 subgroups (Table 2). Moreover, the quality of response did not differ by severity of RI. A VGPR or better was achieved in 34% of patients with mild or no RI, in 27% of patients with moderate RI, and in 38% of patients with severe RI (*P* = not significant [NS] for both). The TTP was similar in patients with mild or no RI (median, 12.0 months) and patients with moderate RI (median, 11.1 months) (Table 2). Patients who had severe RI had a shorter TTP; however, the difference was not significant compared with patients who had mild or no RI (median, 7.8 months; *P* = NS). Similarly, PFS did not differ significantly between patients who had mild or no RI (median, 11.1 months) and patients who had moderate RI (median, 9.5 months; *P* = NS) or severe RI (median, 7.8 months; *P* = NS) (Table 2, Fig. 1).

**Table 2:** Efficacy Outcomes According to Renal Function

	No. of Patients (%)		
Outcome	Mild or No RI: CL_Cr_ ≥60 mL/min	Moderate RI: CL_Cr_ ≥30 mL/min to <60 mL/min	Severe RI: CL_Cr_ <30 mL/min
Total no. of patients	243	82	16
Response			
Overall response	156 (64)	46 (56)	8 (50)
Complete response	38 (16)	13 (16)	1 (6)
Very good partial response	45 (19)	9 (11)	5 (31)
Partial response	73 (30)	24 (29)	2 (13)
Stable disease	69 (28)	28 (34)	5 (31)
Progressive disease	5 (2)	3 (4)	0
Response not evaluable^a^	13 (5)	5 (6)	3 (19)
Efficacy, mo			
Median time to progression	12.0	11.1	7.8
Median PFS	11.1	9.5	7.8
Median OS	38.9	29.0^b^	18.4^b^

RI indicates renal impairment; CL_Cr_, creatinine clearance; PFS, progression-free survival; OS, overall survival.

a *P* = .006 versus patients with mild or no RI.

b Including patients who did not have any response assessment data at the data cutoff point or whose only assessment was “response not evaluable.”

**Figure 2:**
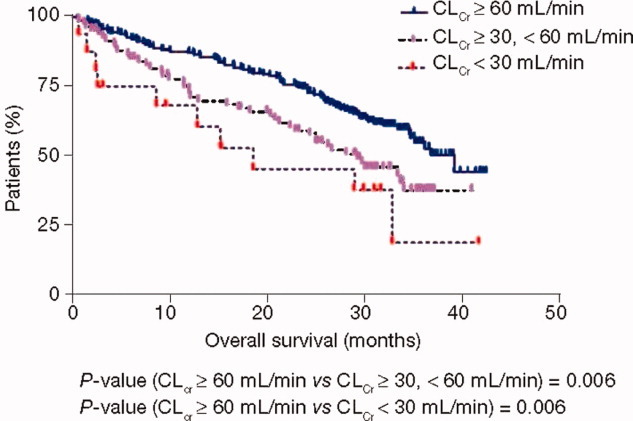
This is a Kaplan-Meier plot of overall survival according to renal impairment (RI). *P* < .01 for patients with severe RI compared with patients with mild or no RI. RI was defined according to the creatinine clearance (CL_Cr_) level as mild or no RI (CL_Cr_ ≥60 mL/minute), moderate RI (CL_Cr_ from ≥30 mL/minute to <60 mL/minute), or severe RI (CL_Cr_ <30 mL/minute).

After a median follow-up of 31.3 months, OS was 38.9 months for patients who had mild or no RI (Table 2). Patients who had moderate or severe RI tended to have shorter OS (29.0 months and 18.4 months, respectively), and this was significantly shorter compared with the OS of patients who had mild or no RI (*P* = .006 for both) (Table 2, Fig. 2).

### Improvement in Renal Function

Improvement in CL_Cr_ by at least 1 level (ie, mild or no RI, moderate RI, or severe RI as described above; see Materials and Methods) was experienced by 68 of 94 patients (72%) with moderate-to-severe RI who received lenalidomide plus dexamethasone; whereas, of all those who received lenalidomide plus dexamethasone, only 3 patients (1%) had a worsening CL_Cr_ by at least 1 level (Table 3).

**Table 3:** Shift in Renal Function With Treatment According to Renal Function

	No. of Patients (%)		
Renal Function at Baseline	Mild or No RI: CL_Cr_ ≥60 mL/min	Moderate RI: CL_Cr_ ≥30 mL/min to <60 mL/min	Severe RI: CL_Cr_ <30 mL/min
Mild or no RI, n=238	235 (99)	1 (0.5)	2 (1)
Moderate RI, n=80	56 (70)^a^	24 (30)	0 (0)
Severe RI, n=14	0 (0)	12 (86)^a^	2 (14)
Total, N=332	291 (88)	37 (11)	4 (1)

RI indicates renal impairment; CL_Cr_, creatinine clearance.

a These patients improved with treatment. The best postbaseline renal responses were used.

### Safety

Neutropenia was the most common grade 3 or 4 hematologic adverse event, and the rates of neutropenia were similar across all subgroups of renal function (32%, 48%, and 38% of patients with mild or no RI, moderate RI, and severe RI, respectively) (Table 4). Renal impairment was not associated with a higher incidence of fever or infections during neutropenia. However, there was an increased incidence of pneumonia unrelated to neutropenia in the limited number of patients with severe RI (Table 4). Thrombocytopenia was significantly more common in patients with severe RI (38%; *P* < .05) and moderate RI (22%; *P* < .05) than in patients with mild or no RI (9%). Grade 3 or 4 thrombotic events occurred in patients with mild or no RI (13%), moderate RI (15%), and severe RI (6%) (Table 4).

**Table 4:** Grade 3 or 4 Adverse Events According to Renal Function That Occurred in >10% of Patients

Adverse Event	Mild or No RI: CL_Cr_ ≥60 mL/min	Moderate RI: CL_Cr_ ≥30 mL/min to <60 mL/min	Severe RI: CL_Cr_ <30 mL/min
Total no. of patients	243	82	16
Hematologic toxicities, %			
Neutropenia	32	48^a^	38
Thrombocytopenia	9	22^a^	38^a^
Anemia	5	21^a^	44^a^
Nonhematologic toxicities, %			
Thrombotic events^b^	13	15	6
Hypertension NOS	0.8	2	13
Atrial fibrillation	3	4	13
Fatigue	5	12^a^	0
Asthenia	4	5	13
Constipation	2	1	13
Hypocalcemia	3	6	19
Dehydration	0.8	2^a^	13^a^
Pneumonia NOS	7	9	25^a^
Clinically important adverse events, %			
Febrile neutropenia	3	2	0
Neuropathy	2	2	0
Peripheral neuropathy	2	1	0

RI indicates renal impairment; CL_Cr_, creatinine clearance; NOS, not otherwise specified.

a *P* < .05 versus patients with mild or no RI.

b Thrombotic events included pulmonary embolism, deep vein thrombosis, and venous thrombosis NOS.

All patients received a starting lenalidomide dose of 25 mg. The median dose of lenalidomide was 25 mg for patients with moderate or better RI and 15 mg for patients with severe RI (Table 5). Significantly more patients with moderate RI (40%) and severe RI (38%) required a lenalidomide dose reduction or interruption because of adverse events than patients with mild or no RI (22%; *P* < .05). The median time to lenalidomide dose reduction was 99 days, 85 days, and 78 days for patients with mild or no RI, moderate RI, and severe RI, respectively. Overall, lenalidomide discontinuation because of adverse events (in almost all patients because of cytopenias) increased with the severity of RI and was significantly more frequent for patients with severe RI (38%) than for patients with mild or no RI (12%; *P* < .05).

**Table 5:** Dosage Information According to Renal Function

Variable	Mild or No RI: CL_Cr_ ≥60 mL/min	Moderate RI: CL_Cr_ ≥30 mL/min to <60 mL/min	Severe RI: CL_Cr_ <30 mL/min
Total no. of patients	243	82	16
Median lenalidomide dose, mg/d	25	25	15^a^
Dose reduction/interruption because of AE, %	22	40^a^	38^a^
Median time to lenalidomide dose reduction (range), d	99 (29-486)	85 (29-561)	78 (29-374)
Discontinuation because of AE, %	12	18	38^a^

RI indicates renal impairment; CL_Cr_, creatinine clearance; AE, adverse event.

a *P* < .05 versus patients with mild or no RI.

## DISCUSSION

Renal impairment is a frequent complication in patients with MM, and its incidence increases in patients with recurrent disease and as the disease becomes refractory to treatment.^1^ Two large, prospective randomized trials, MM-009 and MM-010, established the significant activity of combination lenalidomide plus dexamethasone in patients with relapsed and/or refractory MM.^12^,^13^ Although lenalidomide is excreted renally, many patients with advanced myeloma and RI may not have any other treatment option. This frequent clinical complication prompted a retrospective analysis of data from MM-009 and MM-010 to evaluate the effects of RI on patient outcomes. The results from the current subgroup analysis demonstrate that the combination of lenalidomide plus dexamethasone is effective in patients with all degrees of renal dysfunction. Renal impairment at the time of diagnosis is associated with poor survival in patients with MM.^6^,^9^ Persistent renal failure is associated with an increased risk of morbidity and early mortality.^18^,^19^ In 1 study, the median survival was an additional 6 months for patients who had reversible renal failure compared with patients who had irreversible renal injury, indicating that renal improvement may be associated with improved long-term survival.^6^ Thus, the reversibility of renal failure may be an important prognostic factor, perhaps as important as quality of response to therapy, for patients with RI.^6^,^9^ The improvement in RI to a near-normal range (CL_Cr_ ≥60 mL/minute) observed in the majority of patients in the current study suggests that lenalidomide plus dexamethasone may be a particularly useful therapy in this setting.

In the current study, TTP and PFS did not differ significantly between patients who had mild or no RI compared with patients who had RI. However, patients who had severe RI (CL_Cr_ <30 mL/minute) tended to have shorter PFS and a trend toward shorter TTP. Patients who had moderate or severe RI had significantly shorter OS (29.0 months and 18.4 months, respectively) compared with patients who had mild or no RI (38.9 months; *P* = .006 for both). Furthermore, more patients who had severe RI discontinued therapy because of adverse events (predominantly cytopenias). These differences may be associated with treatment schedule: Patients with moderate RI received a median daily dose of 25 mg, similar to patients with mild or no RI, and patients with severe RI received a median daily dose of 15 mg; thus, patients with severe RI may have received longer treatment and greater benefit at a lower starting and treatment dose, consistent with the recent dosing adjustment recommendations for this patient population (Table 6).

**Table 6:** Recommendations for Lenalidomide Dosing in Patients With Multiple Myeloma Who Have Renal Impairment^a^

Category	Renal Function^b^	Lenalidomide Dosing in Multiple Myeloma
Moderate RI	CL_Cr_ ≥30 mL/min to <60 mL/min	10 mg every 24 h
Severe RI	CL_Cr_ <30 mL/min (not requiring dialysis)	15 mg every 48 h
End-stage renal disease	CL_Cr_ <30 mL/min (requiring dialysis)	5 mg once daily; on dialysis days, dose should be administered after dialysis

RI indicates renal impairment; CL_Cr_, creatinine clearance.

a See Celegene Corporation 2009.^20^,^21^

b Cockcroft-Gault CL_Cr_.

The time to dose reduction appeared to be shorter for patients who had RI compared with patients who had mild or no RI, and those who had moderate or severe RI required significantly more frequent intervention because of anemia and thrombocytopenia than those with mild or no RI. Although thrombocytopenia was more pronounced, there were no bleeding events. Other adverse events generally occurred with similar frequencies in patients with and without RI.

The current study highlights the limitations of using the serum creatinine level as a measure of patient renal function, because a subset of eligible patients (serum creatinine <2.5 mg/dL) had severe RI (CL_Cr_ <30 mL/minute) and a greater frequency of certain treatment-related adverse events. Because renal clearance may be reduced by 35% to 50% without evidence of renal disease in elderly patients, dosage adjustments are necessary for renally excreted drugs. In addition, because creatinine production is lower in elderly individuals due to decreased muscle mass and because serum creatinine may be normal despite impaired CL_Cr_, it is recommended to base dosage adjustments on CL_Cr_ instead of serum creatinine (Table 6).^20^ By using CL_Cr_ as a guide for dosing patients with MM who have RI, Chen and colleagues^14^ studied the effect of RI on the pharmacokinetics of lenalidomide after a single 25-mg oral dose (patients aged 39-76 years) in patients without cancer. Those authors demonstrated that, in study participants who had increased baseline RI, renal lenalidomide clearance decreased substantially, prolonging its half-life by approximately 6 to 12 hours.^14^ An 80% decrease in lenalidomide clearance was observed in patients with RI compared with healthy individuals, and that decrease corresponded to the primary renal route of drug excretion.^14^ Since this analysis, adjustments to the initial dose of lenalidomide have been recommended in patients with moderate RI (CL_Cr_ ≥30 mL/minute and <60 mL/minute) or severe RI (CL_Cr_ <30 mL/minute not requiring dialysis) and end-stage renal failure (CL_Cr_ ≥30 mL/minute and <60 mL/minute requiring dialysis). On the basis of a pharmacokinetic study in patients who had RI caused by nonmalignant conditions, the recommendations for the starting dose of lenalidomide in patients with MM who have RI to maintain appropriate exposure are shown in Table 6.^20^,^21^

In conclusion, lenalidomide plus dexamethasone led to improvement in RI in the majority of patients in the current analysis. With careful monitoring of CL_Cr_ levels and adverse events and with appropriate dose adjustments, lenalidomide plus dexamethasone is a highly effective treatment and is well tolerated in patients with MM who have RI. Platelet counts also should be monitored carefully, because we observed more marked thrombocytopenia in patients who had a CL_Cr_ <30 mL/minute, although there did not appear to be any increased incidence of bleeding in the 16 patients in the current study. Formal studies confirming the efficacy of lenalidomide in patients with renal failure are warranted and ongoing. Currently, 2 trials are recruiting for the study of lenalidomide treatment in patients with MM who have RI. These include a study of the pharmacokinetics of lenalidomide in patients with MM who have RI (National Clinical Trial [NCT] 00779922) and a phase 1/2 trial of lenalidomide plus low-dose dexamethasone in patients with relapsed and/or refractory MM and RI (NCT00790842). For future studies and therapy with lenalidomide, it is important to convert serum creatinine to CL_Cr_ and to use CL_Cr_ for dosage adjustments, as recommended in Table 6, for patients with RI.
